# Supplementary material for: Strain engineering of two-dimensional multilayered heterostructures for beyond-lithium-based rechargeable batteries
Source: Nat Commun. 2020 Jul 3;11:3297. doi: 10.1038/s41467-020-17014-w (PMC7335097; doi:10.1038/s41467-020-17014-w)
Supplement: Supplementary file 1 — Supplementary Information [file 41467_2020_17014_MOESM1_ESM.pdf]

Supplementary Information

Strain engineering of two-dimensional multilayered heterostructures for beyond-lithium based rechargeable batteries

Xiong *et al.*

Supplementary Figures

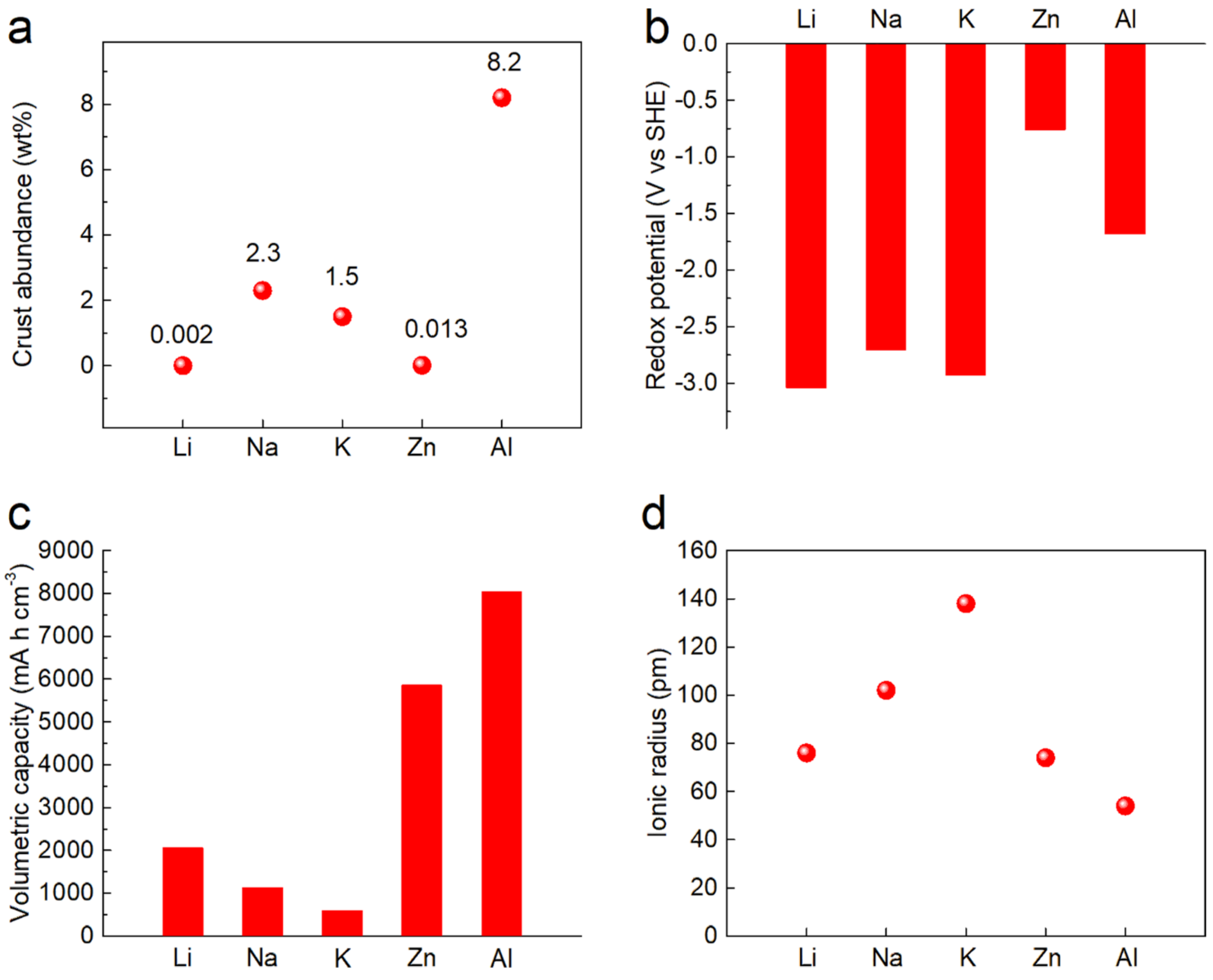

Supplementary Figure 1. Comparison between Li, Na, K, Zn, and Al ions in terms of **a** crust abundance, **b** redox potential, **c** theoretical volumetric capacity and **d** ionic radius.

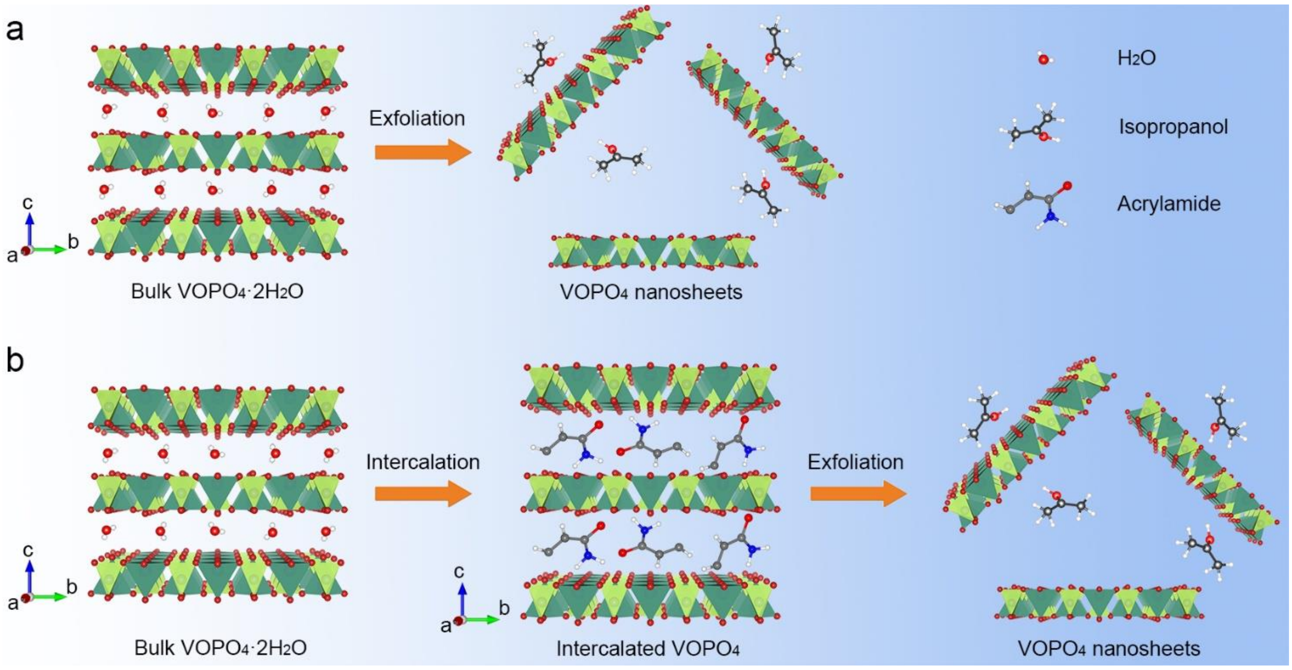

Supplementary Figure 2. Synthesis of $VOPO_4$ nanosheets. **a** The direct exfoliation method. The layered $VOPO_4{\cdot}2H_2O$ crystals were directly exfoliated in isopropanol. **b** An intercalation-exfoliation strategy. The layered $VOPO_4{\cdot}2H_2O$ crystals were first intercalated with acrylamide. The expanded $VOPO_4$-acrylamide compounds were then exfoliated in isopropanol.

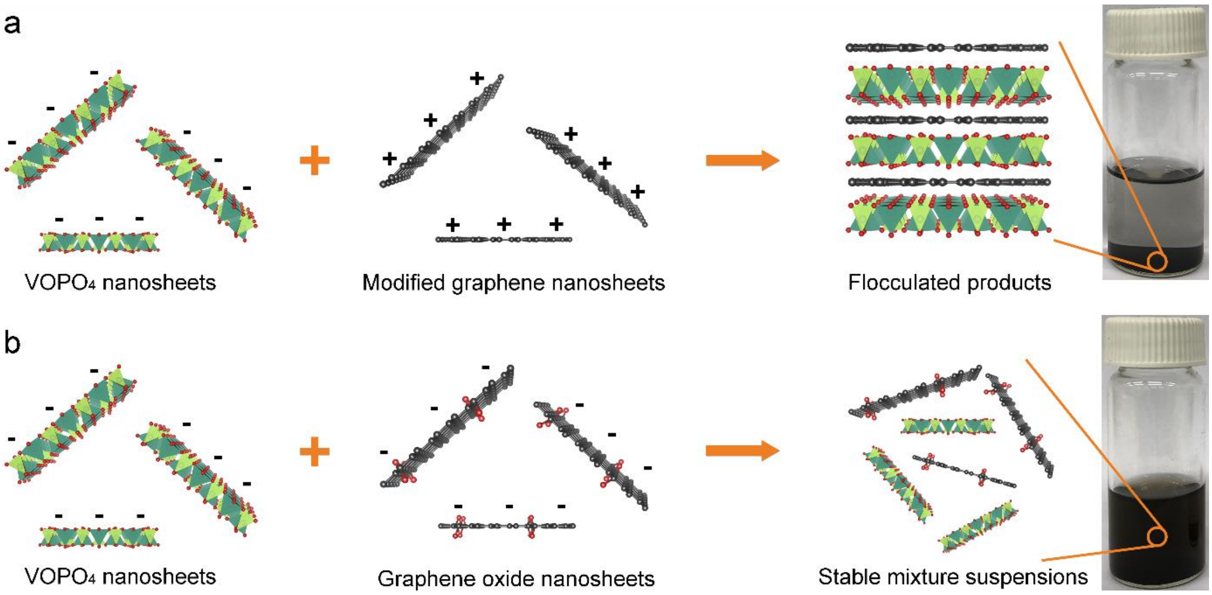

Supplementary Figure 3. Mixing of $VOPO_4$ and graphene. **a** Flocculated products were obtained after mixing negatively charged $VOPO_4$ nanosheets and modified graphene nanosheets with a positively charged nature. **b** A stable mixture suspension was obtained after mixing $VOPO_4$ and graphene oxide nanosheets, which are both negatively charged.

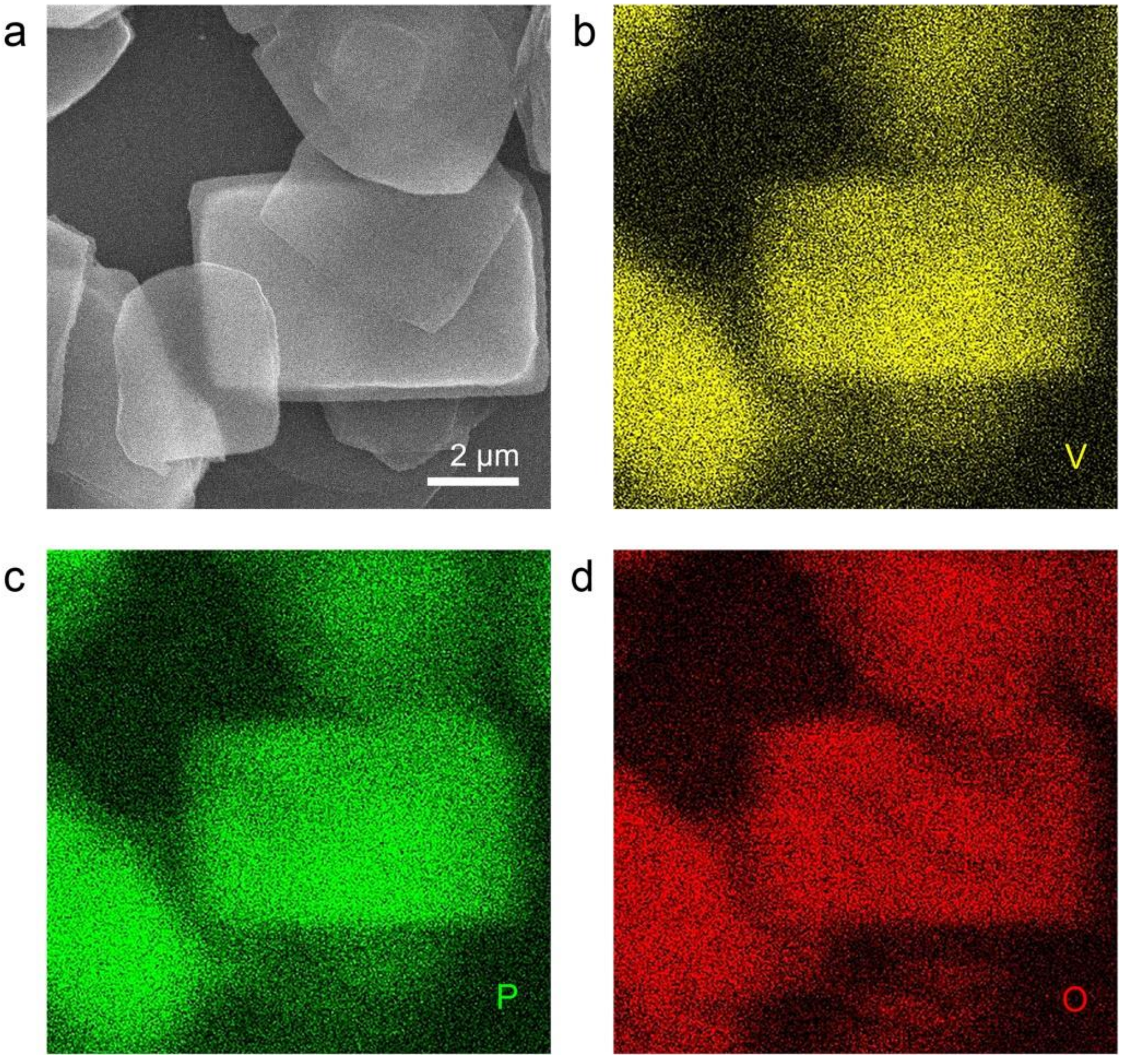

Supplementary Figure 4. Characterization of bulk layered $VOPO_4 \cdot 2H_2O$ crystals. **a** SEM image and **b-d** corresponding element mapping of bulk layered $VOPO_4 \cdot 2H_2O$ crystals.

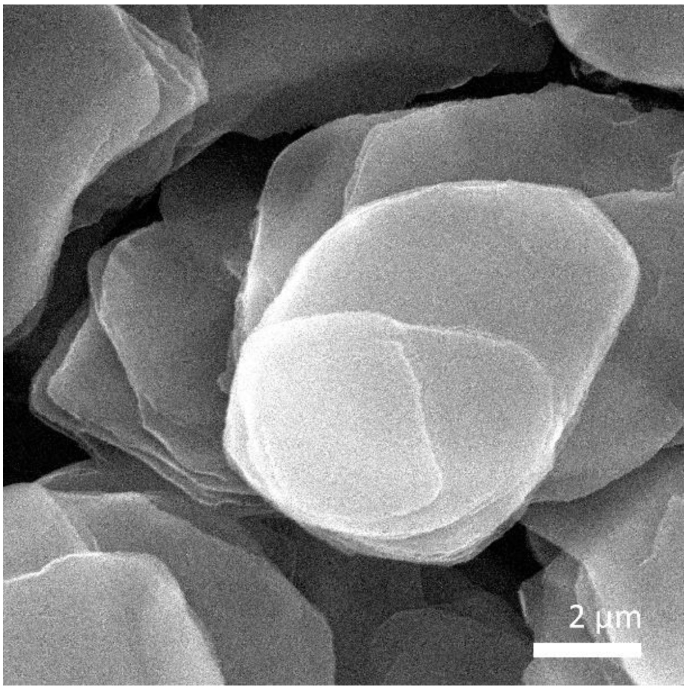

Supplementary Figure 5. SEM image of intercalated $VOPO_4$-acrylamide compounds.

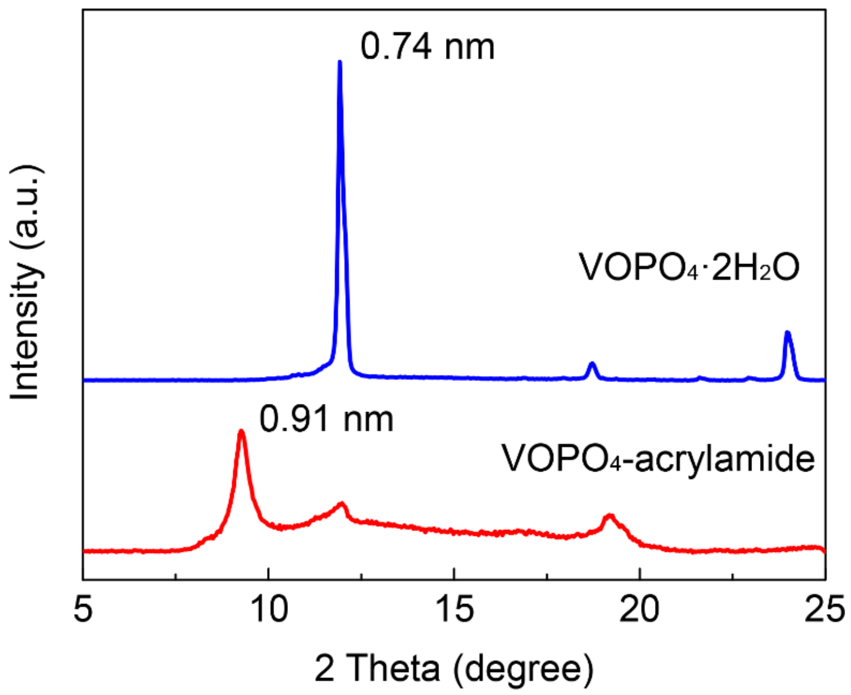

Supplementary Figure 6. XRD patterns of bulk $VOPO_4 \cdot 2H_2O$ and intercalated $VOPO_4$-acrylamide compounds.

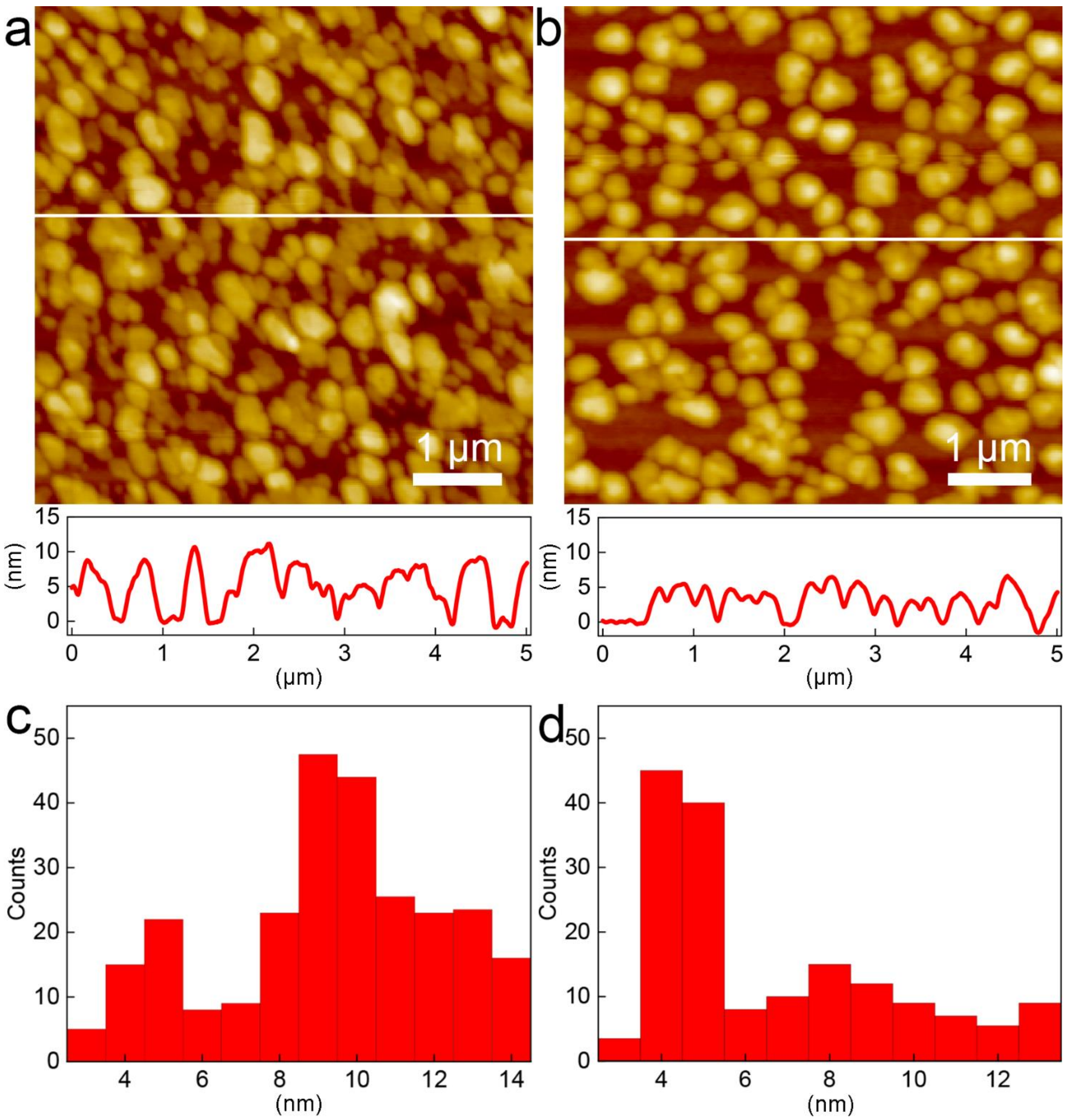

Supplementary Figure 7. AFM images and corresponding thickness profile of $VOPO_4$ nanosheets prepared by **a** the direct exfoliation method and **b** the intercalation-exfoliation strategy. Thickness distribution of the exfoliated VOPO4 nanosheets prepared by **c** the direct exfoliation method and **d** the intercalation-exfoliation strategy.

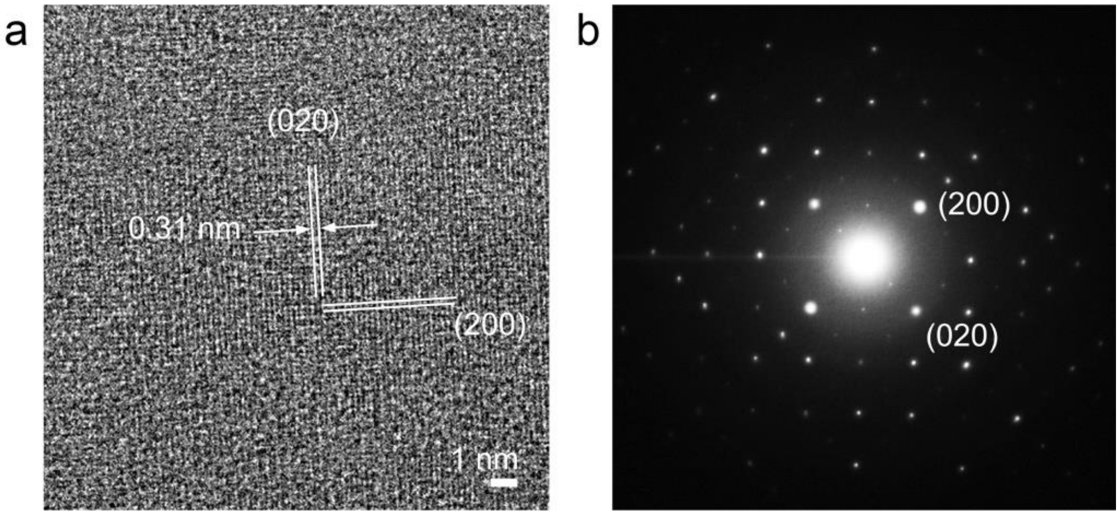

Supplementary Figure 8. Characterization of $VOPO_4$ nanosheets. **a** HRTEM image and **b** selected area electron diffraction (SAED) pattern of exfoliated $VOPO_4$ nanosheets.

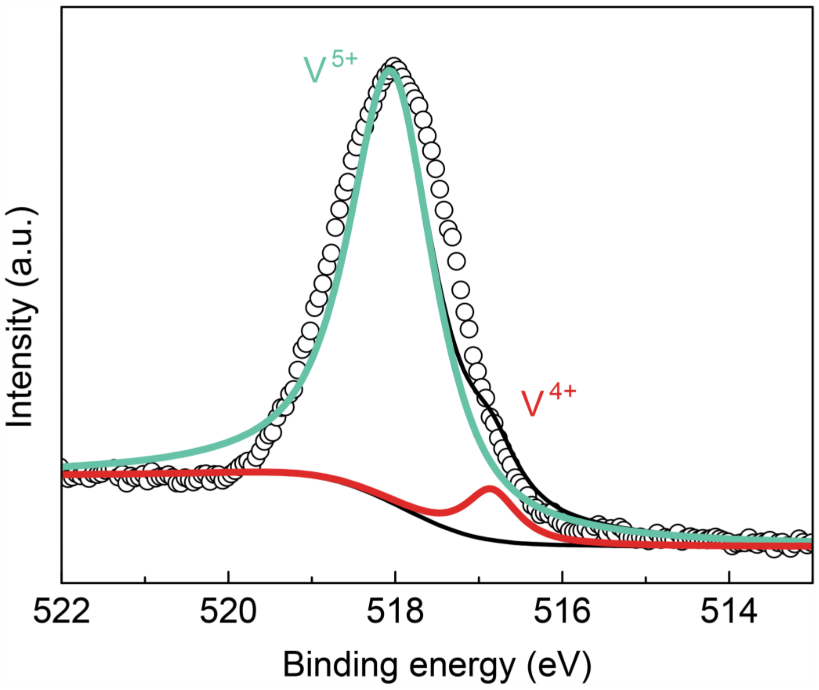

Supplementary Figure 9. The high-resolution spectra of V 2p in exfoliated $VOPO_4$ nanosheets. The reduction of V^{5+} to V^{4+} was observed after the the exfoliation process, leading to the formation of a negatively charged nature of the $VOPO_4$ nanosheets.

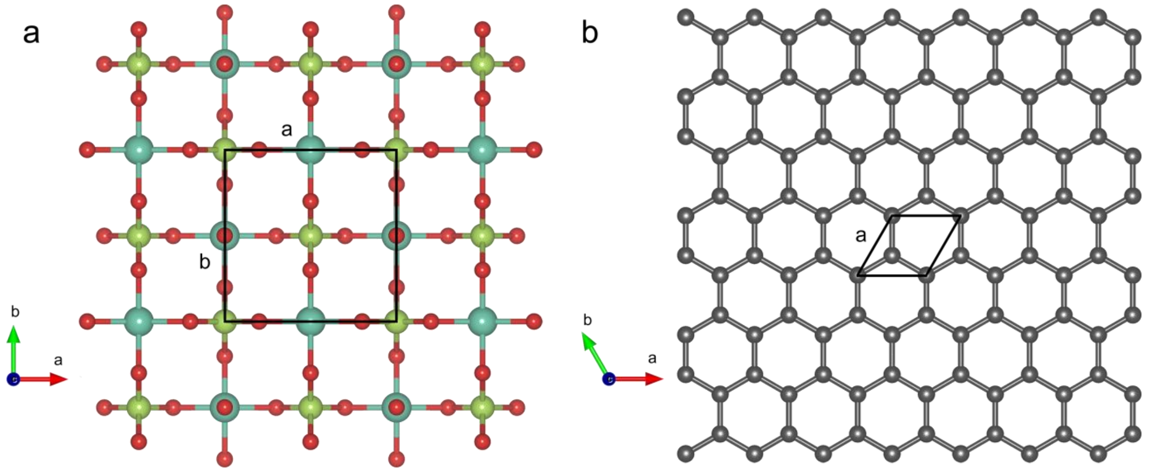

Supplementary Figure 10. The $VOPO_4$ and modified graphene nanosheets were face-to-face restacked in the $VOPO_4$-graphene multilayered heterostructures. The mixing ratio between these two nanosheets could be theoretically calculated based on a hypothesized area-matching model using the in-plane unit cell area of both nanosheets. For an approximate calculation, the ideal graphene structure was used to estimate the area matching between modified graphene and $VOPO_4$. **a** In-plane structure of $VOPO_4$ with a cubic unit cell: $a = b = 0.62$ nm. **b** In-plane structure of graphene with a hexagonal unit cell: $a = 0.25$ nm. The 2D weight density of graphene was $W_{(graphene)} = 2\ M_{(C)} / (a \times a \times \sin 120° \times N_A)$, and the 2D weight density of $VOPO_4$ monolayer was $W_{(VOPO4)} = 2\ M_{(VOPO4)} / (a \times b \times N_A)$, where N_A is the Avogadro's number and $M_{(C)}$ and $M_{(VOPO4)}$ are the formula weights of carbon and $VOPO_4$, respectively. The mass ratio between $VOPO_4$ monolayer and modified graphene under the area balance 1: 1 is $m_{(VOPO4)} / m_{(graphene)} = W_{(VOPO4)} / W_{(graphene)} = \sim 1.9$. The exfoliated $VOPO_4$ nanosheet with a thickness of ~4.0 nm was comprised of around 6 monolayers. Accordingly, the mass ratio between $VOPO_4$ nanosheets and modified graphene in the $VOPO_4$-graphene multilayered heterostructures could be estimated as ~11.4.

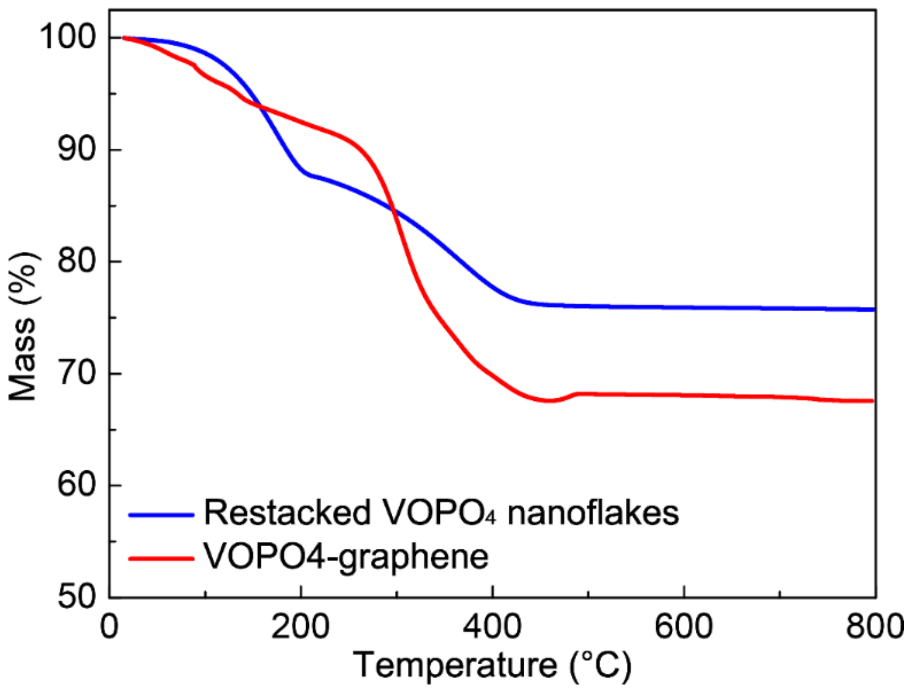

Supplementary Figure 11. TG analysis of restacked $VOPO_4$ nanoflakes and $VOPO_4$-graphene multilayered heterostructures. The weight loss of the restacked $VOPO_4$ nanoflakes is ~25%, which is attributed to the remove of interlayer isopropanol molecules. The weight loss of $VOPO_4$-graphene is ~33%, which can be attributed to the decomposition of isopropanol molecules and the combustion of graphene. So, the content of modified graphene in the $VOPO_4$-graphene is estimated to be ~10 wt%.

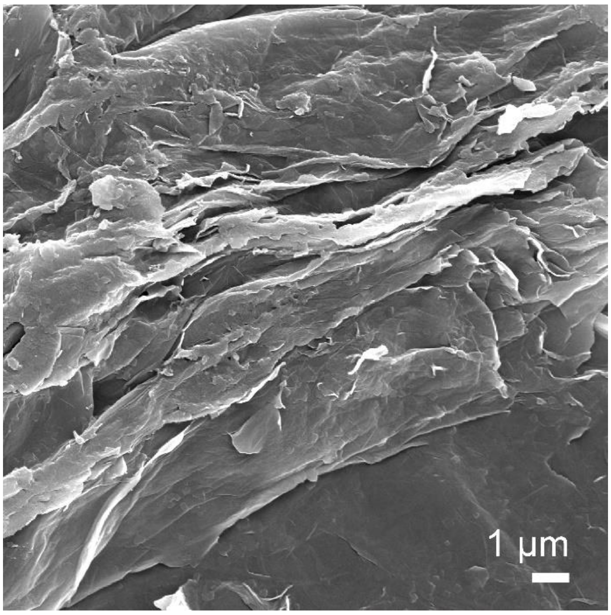

Supplementary Figure 12. A side-view SEM image of $VOPO_4$-graphene showing the multilayered heterostructure morphology.

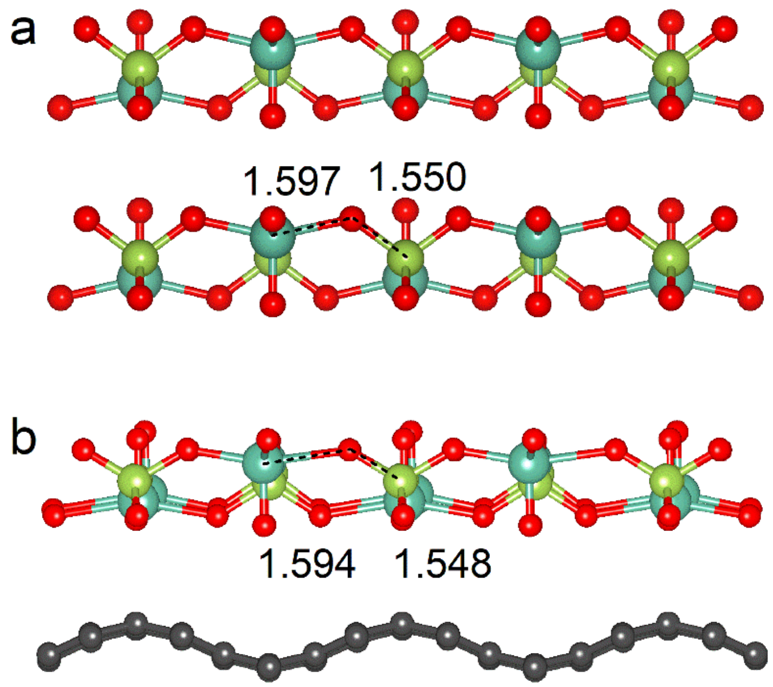

Supplementary Figure 13. Chemical models of the **a** $VOPO_4$-$VOPO_4$ and **b** $VOPO_4$-graphene.

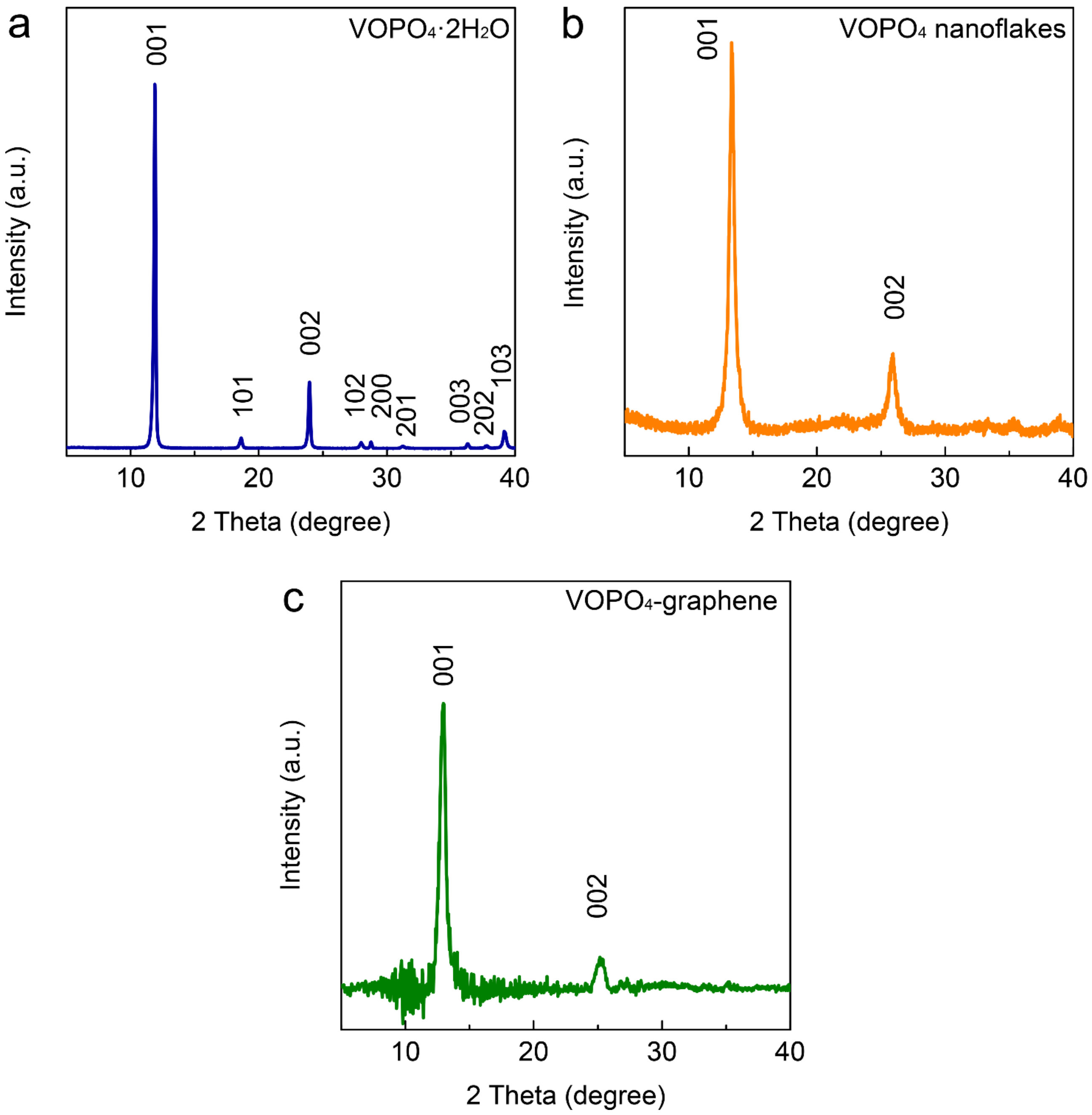

Supplementary Figure 14. XRD patterns of **a** bulk layered $VOPO_4 \cdot 2H_2O$ crystals, **b** restacked $VOPO_4$ nanoflakes and **c** $VOPO_4$-graphene multilayered heterostructures.

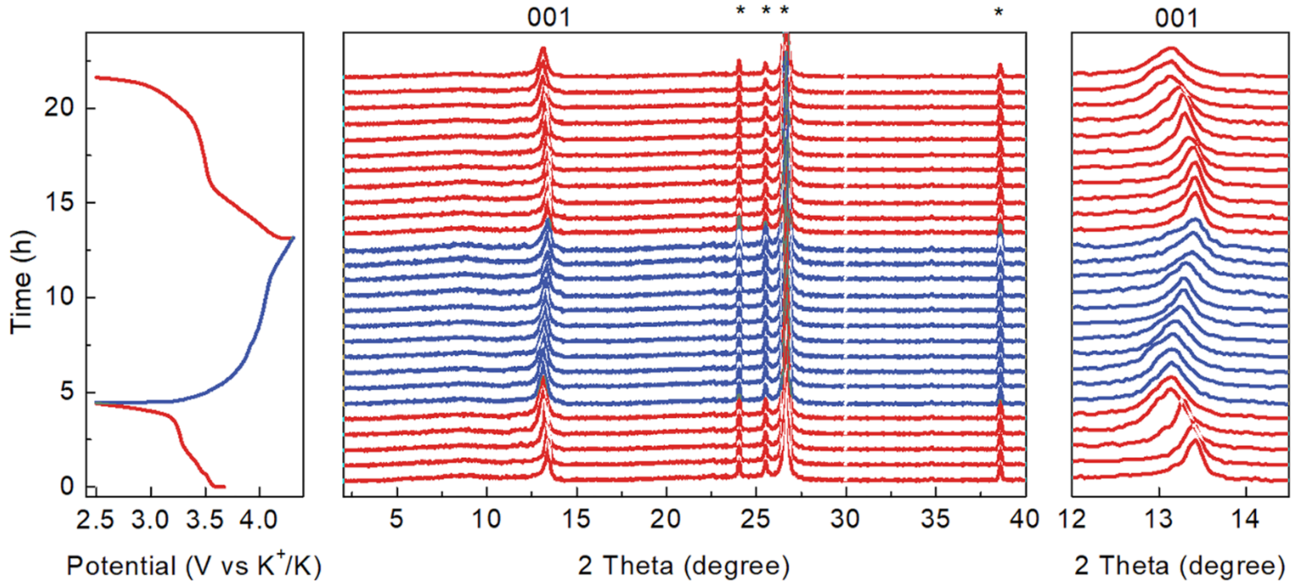

Supplementary Figure 15. The in situ XRD measurements of $VOPO_4$-graphene cathode during the initial charge/discharge cycles. The 001 peaks were observed during the whole charge/discharge cycles. The peaks numbered with * were diffraction signals from the carbon-coated Al foils.

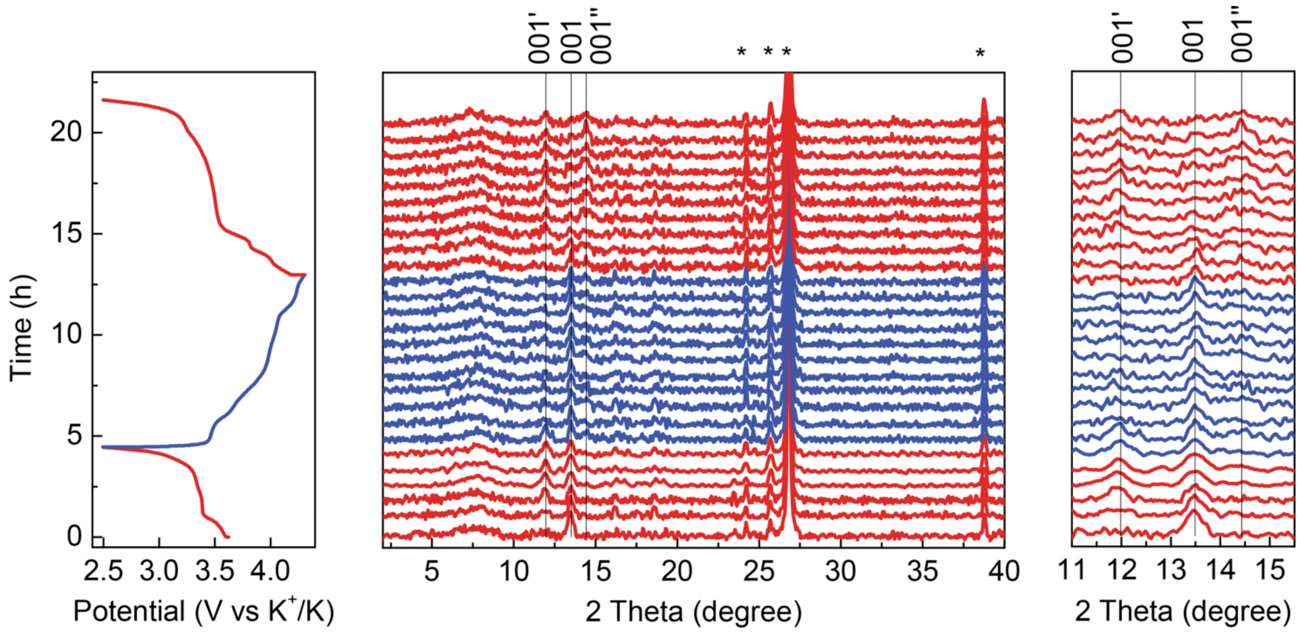

Supplementary Figure 16. The in situ XRD measurements of restacked $VOPO_4$ nanoflakes during the initial charge/discharge cycles. The 001 peaks were disappeared after the charge/discharge cycles. New 001' and 001'' peaks corresponding to new formed phases with different interlayer distances were observed. The peaks numbered with * were diffraction signals from the carbon-coated Al foils.

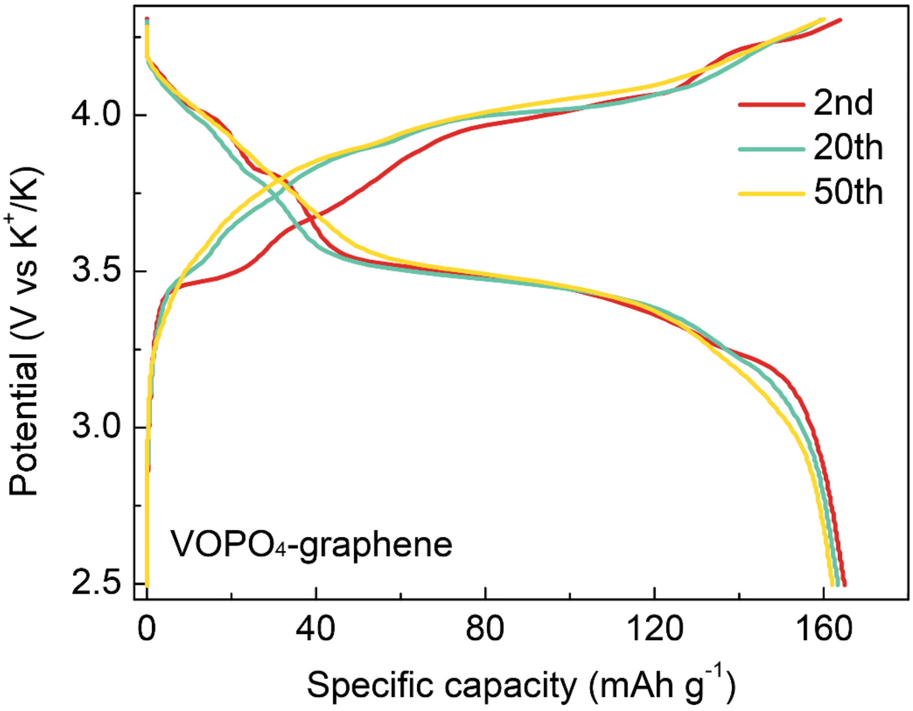

Supplementary Figure 17. The charge/discharge profiles of $VOPO_4$-graphene multilayer heterostructures at the 2nd, 20th and 50th cycles. In addition to no obvious capacity decay, the average discharge plateau of the $VOPO_4$-graphene was still sustained on ~3.5 V.

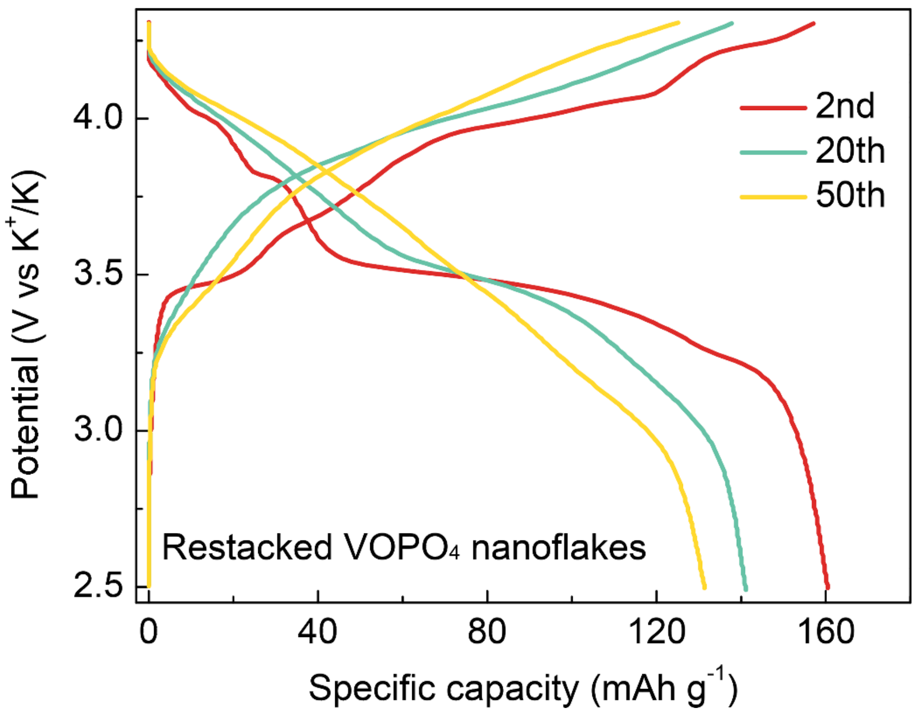

Supplementary Figure 18. The charge/discharge profiles of restacked $VOPO_4$ nanflakes at the 2nd, 20th and 50th cycles. In addition to obvious capacity decay, the polarization of restacked $VOPO_4$ nanoflakes significantly increased and the discharge plateau even vanished after the initial several cycles.

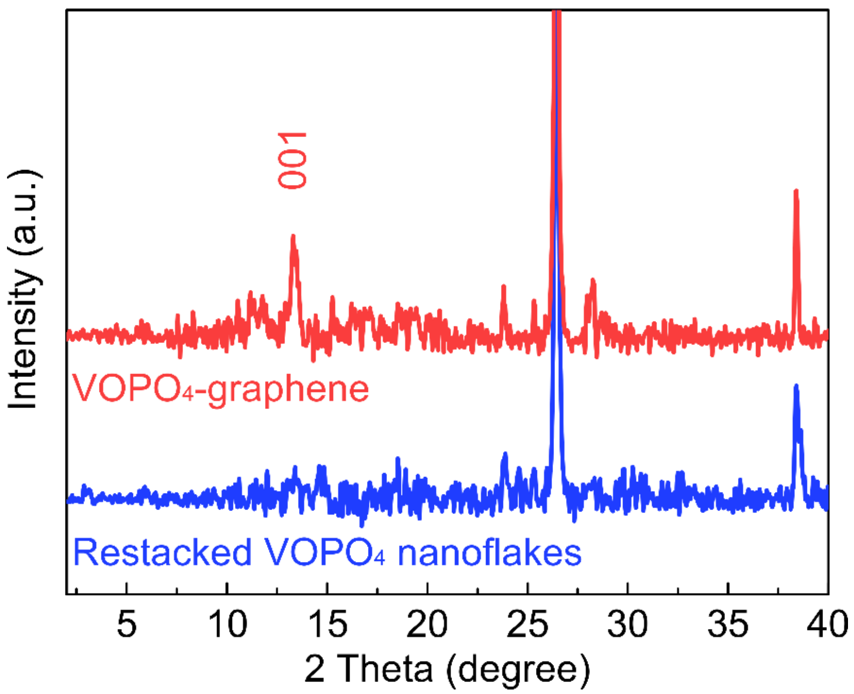

Supplementary Figure 19. XRD patterns of restacked $VOPO_4$ nanoflakes and $VOPO_4$-graphene multilayered heterostructures after 100 charge/discharge cycles. The (001) peak was clearly observed in the $VOPO_4$-graphene multilayered heterostructures, implying the well-maintained layered structures. However, no peaks of the layered structure were shown in the restacked $VOPO_4$ nanoflakes, suggesting the collapse of layered structures after the repeated insertion/extraction of K^+ ions.

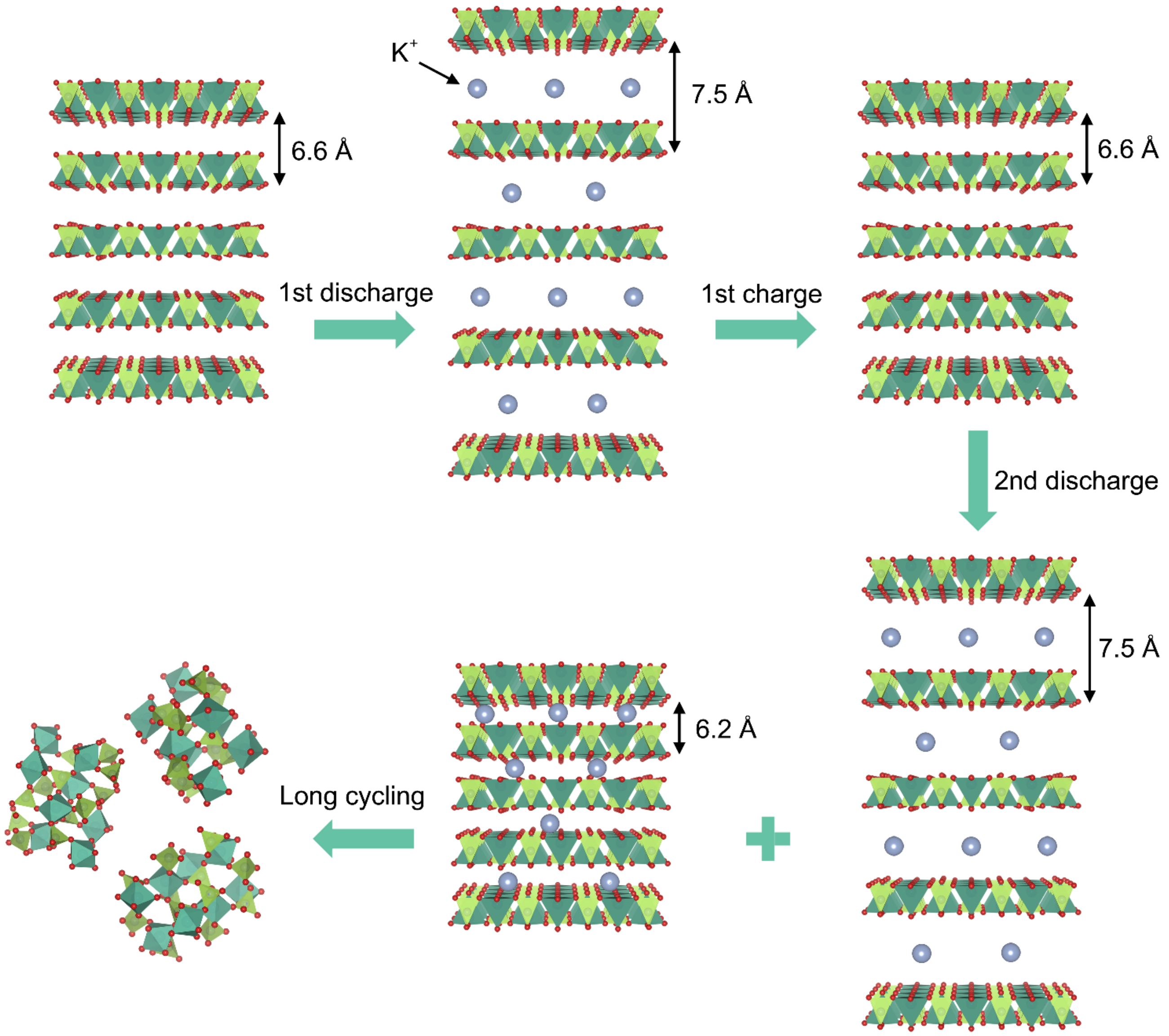

Supplementary Figure 20. Schematic illustration of the potassiation/depotassiation process of restacked $VOPO_4$ nanoflakes during the charge/discharge cycles. The restacked $VOPO_4$ nanoflakes collapse after the repeated insertion/extraction of K^+ ions.

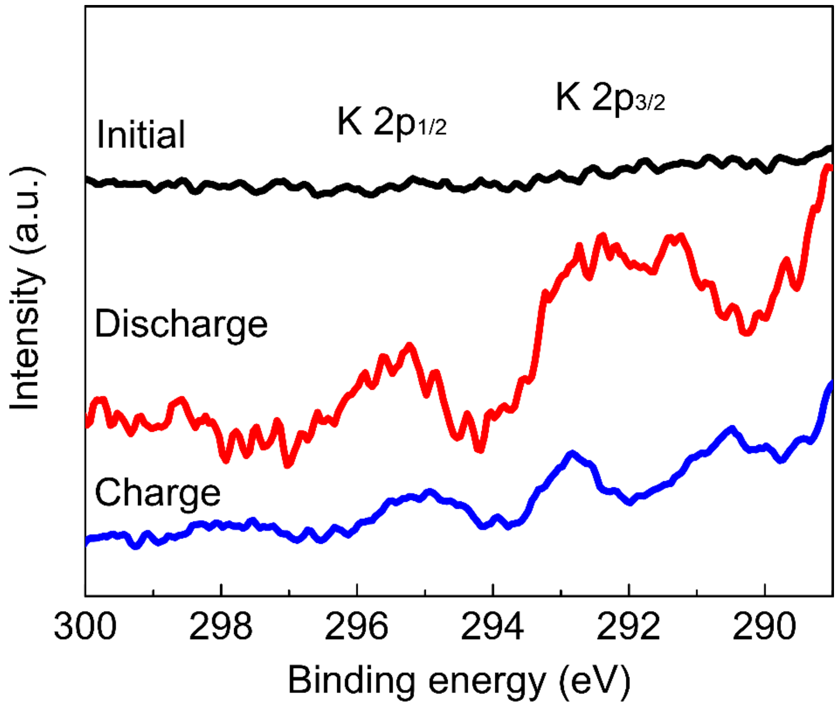

Supplementary Figure 21. Ex-situ K 2p XPS of $VOPO_4$-graphene multilayered heterostructures in initial, discharged and charged states.

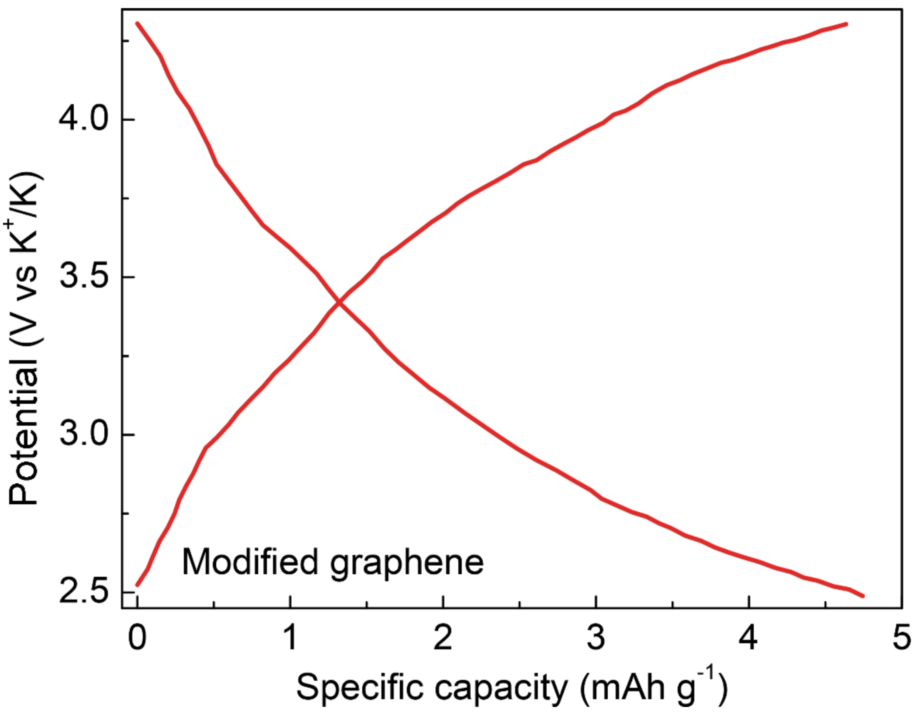

Supplementary Figure 22. The charge/discharge profiles of modified graphene.

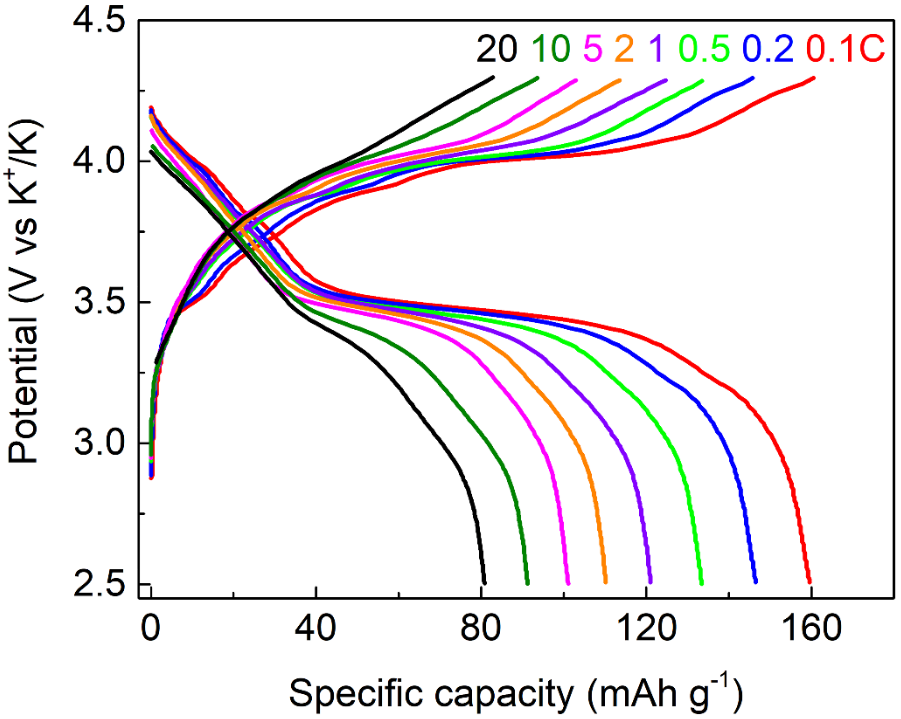

Supplementary Figure 23. The charge/discharge profiles of $VOPO_4$-graphene multilayered heterostructures as cathodes for K-ion batteries at various current densities of 0.1, 0.2, 0.5, 1, 2, 5, 10, and 20 C.

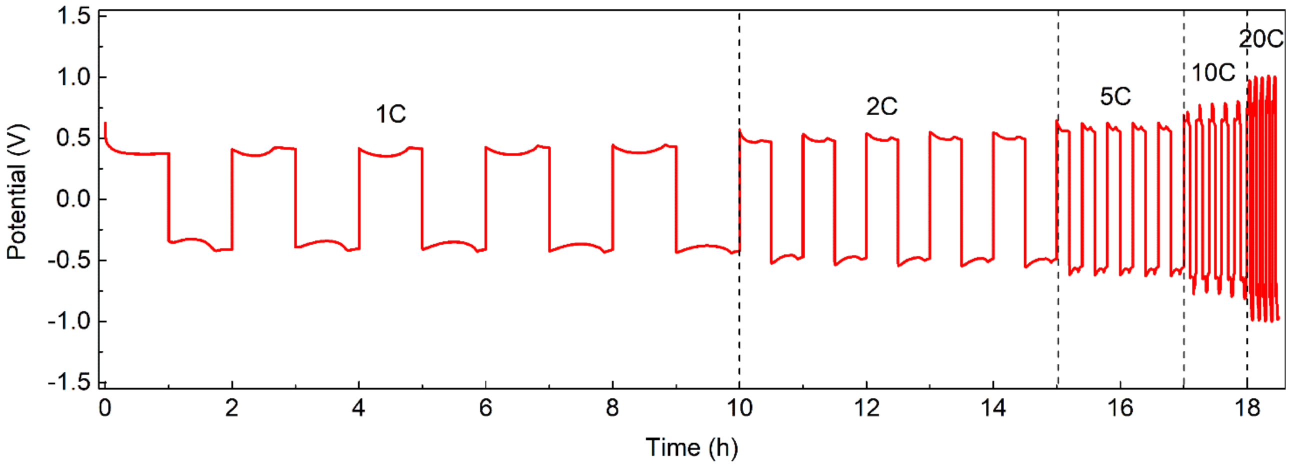

Supplementary Figure 24. Rate performances of symmetric K-K cells at different current densities.

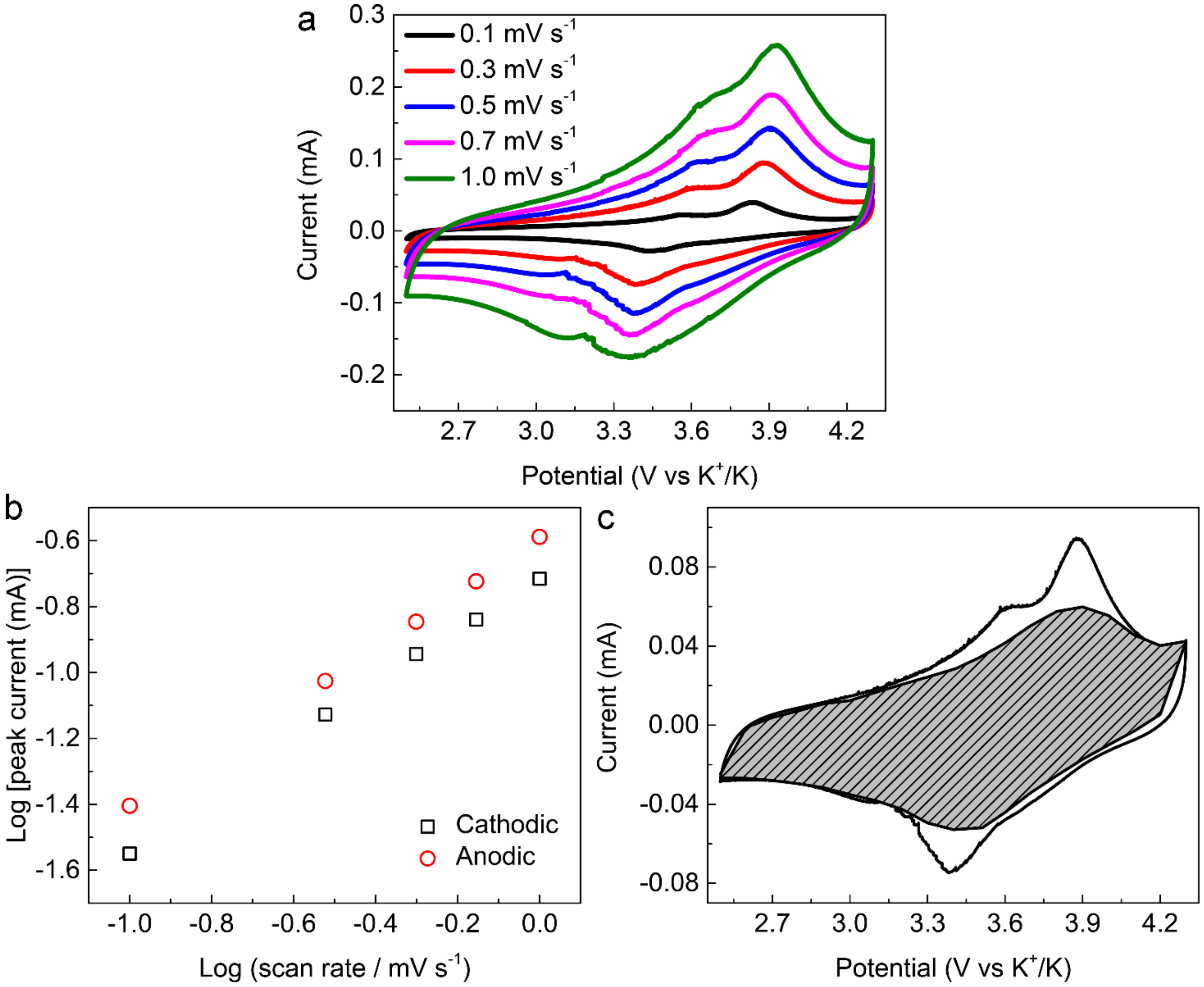

Supplementary Figure 25. Kinetics analysis. **a** CV curves of $VOPO_4$-graphene multilayered heterostructures as cathodes for K-ion batteries at various scan rates. **b** Determination of the *b*-value using the relationship between peak current and scan rate. **c** Separation of the capacitive and diffusion currents at a scan rate of 0.3 mV s^{-1}.

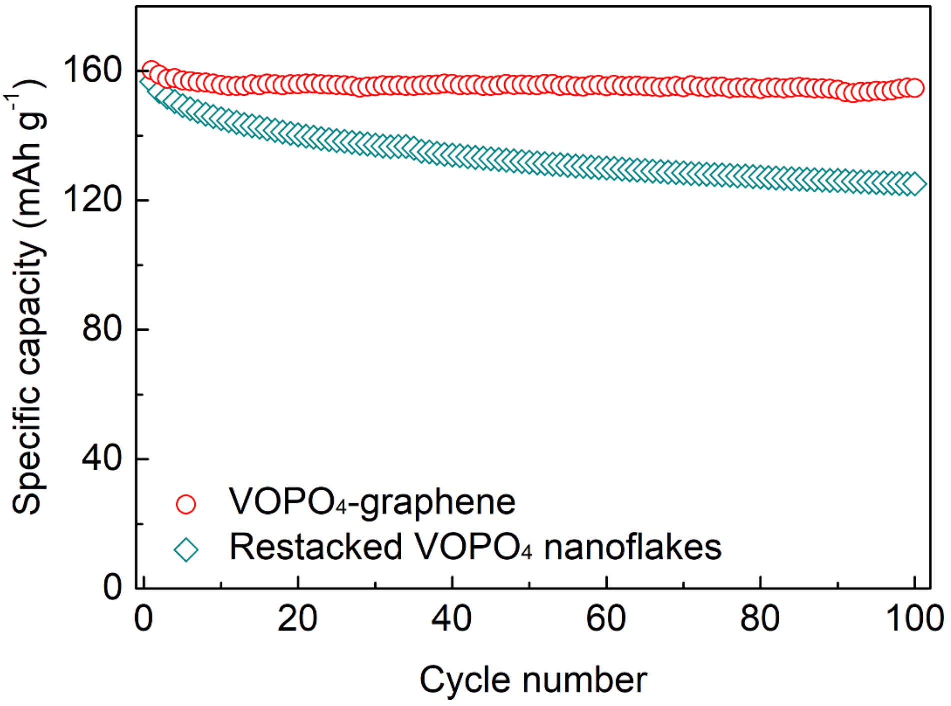

Supplementary Figure 26. Comparison of cycling performance of restacked $VOPO_4$ nanoflakes and $VOPO_4$-graphene as cathodes for Na-ion batteries at 0.1C.

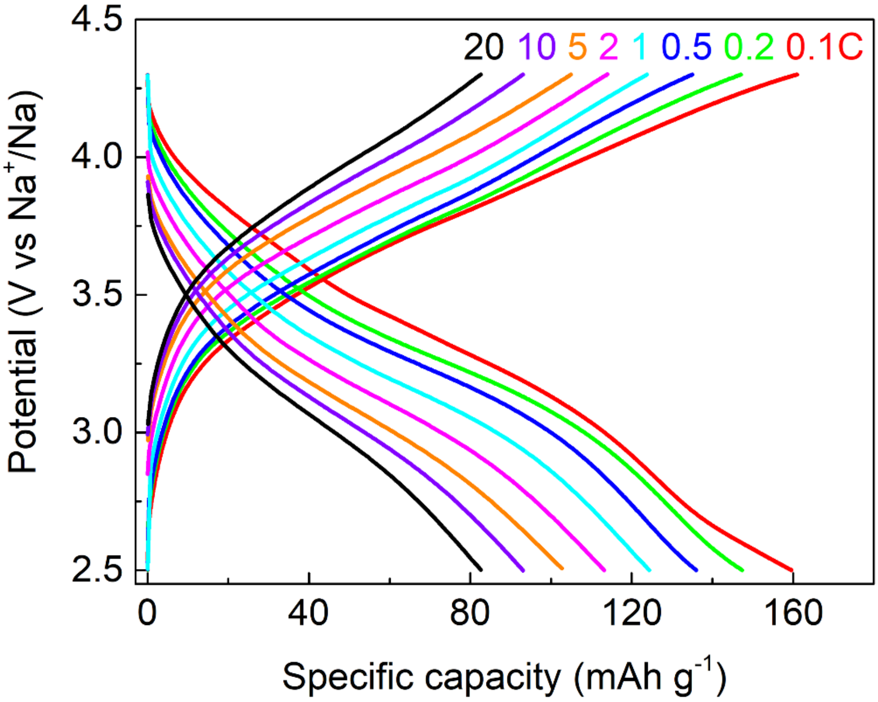

Supplementary Figure 27. The charge/discharge profiles of $VOPO_4$-graphene multilayered heterostructures as cathodes for Na-ion batteries at various current densities of 0.1, 0.2, 0.5, 1, 2, 5, 10, and 20 C.

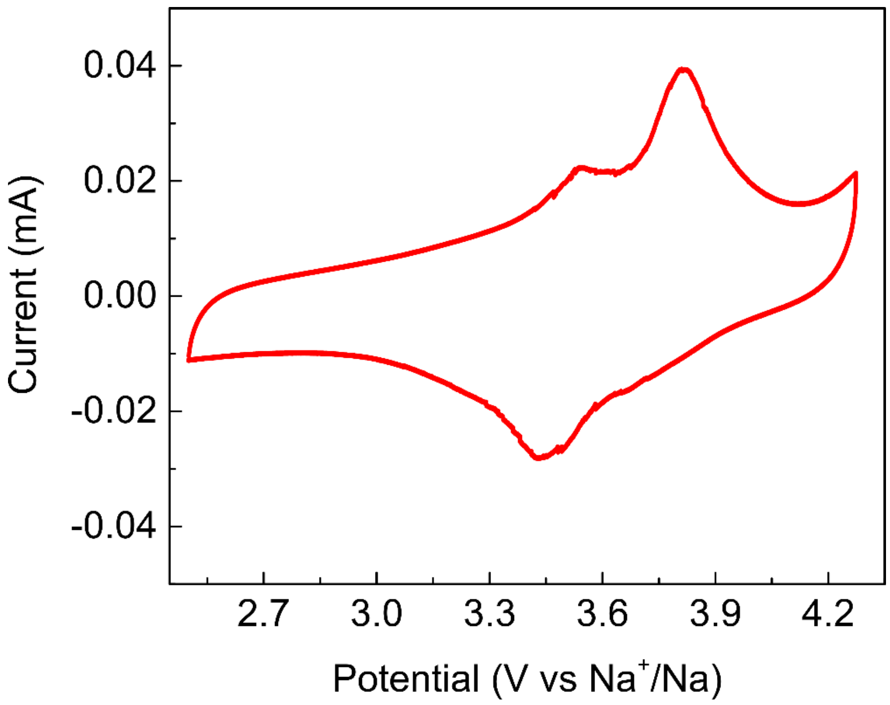

Supplementary Figure 28. CV curves of $VOPO_4$-graphene multilayered heterostructures as cathodes for Na-ion batteries at 0.1 mV s^{-1}.

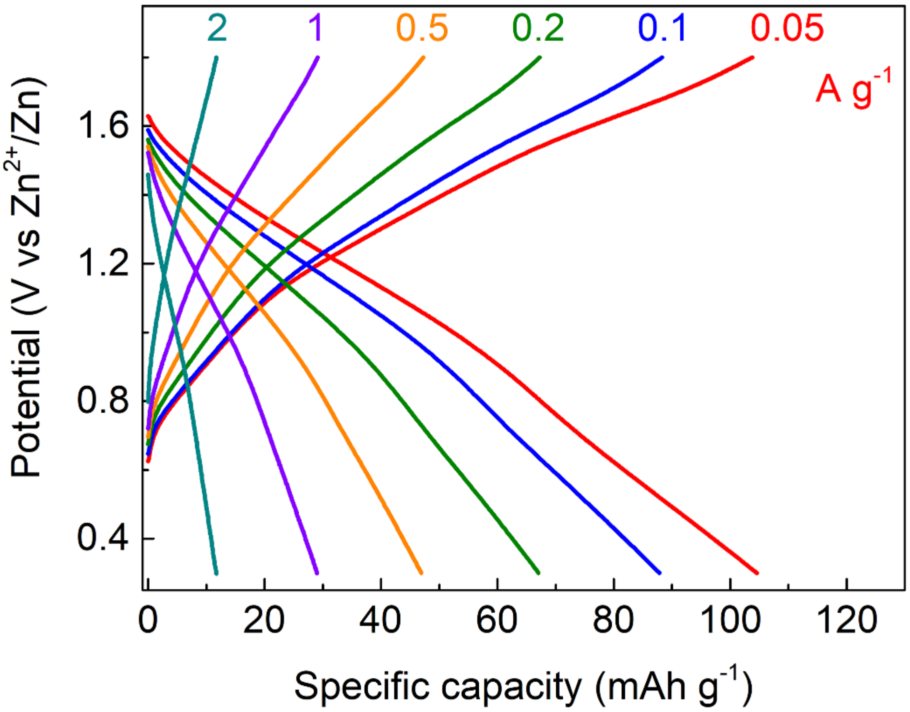

Supplementary Figure 29. The charge/discharge profiles of restacked $VOPO_4$ nanoflakes as cathodes for Zn-ion batteries at various current densities of 0.05, 0.1, 0.2, 0.5, 1, and 2 A g^{-1}.

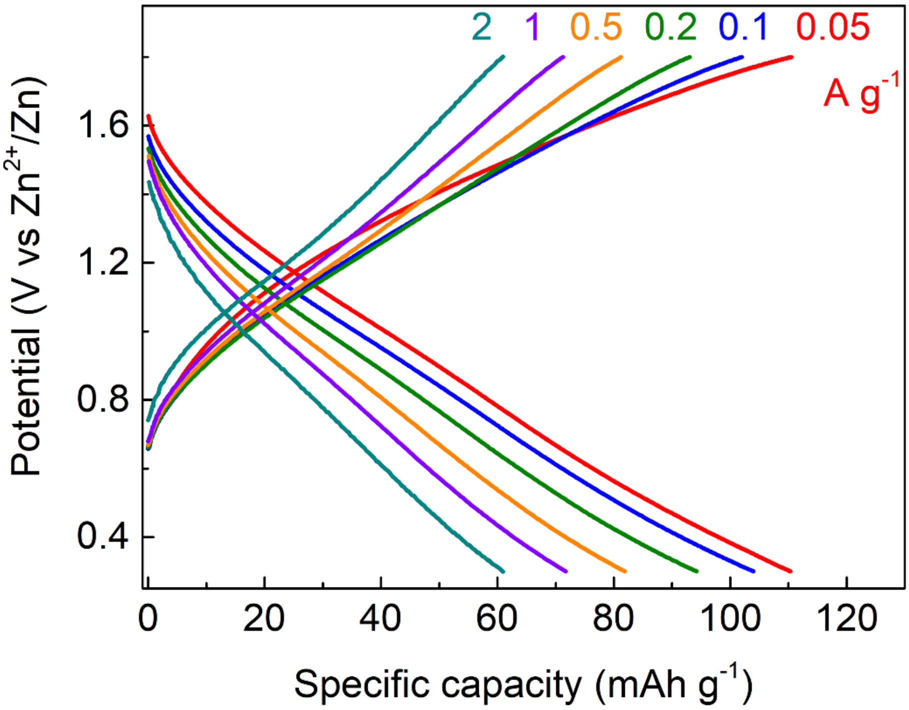

Supplementary Figure 30. The charge/discharge profiles of $VOPO_4$-graphene multilayered heterostructures as cathodes for Zn-ion batteries at various current densities of 0.05, 0.1, 0.2, 0.5, 1, and 2 A g^{-1}.

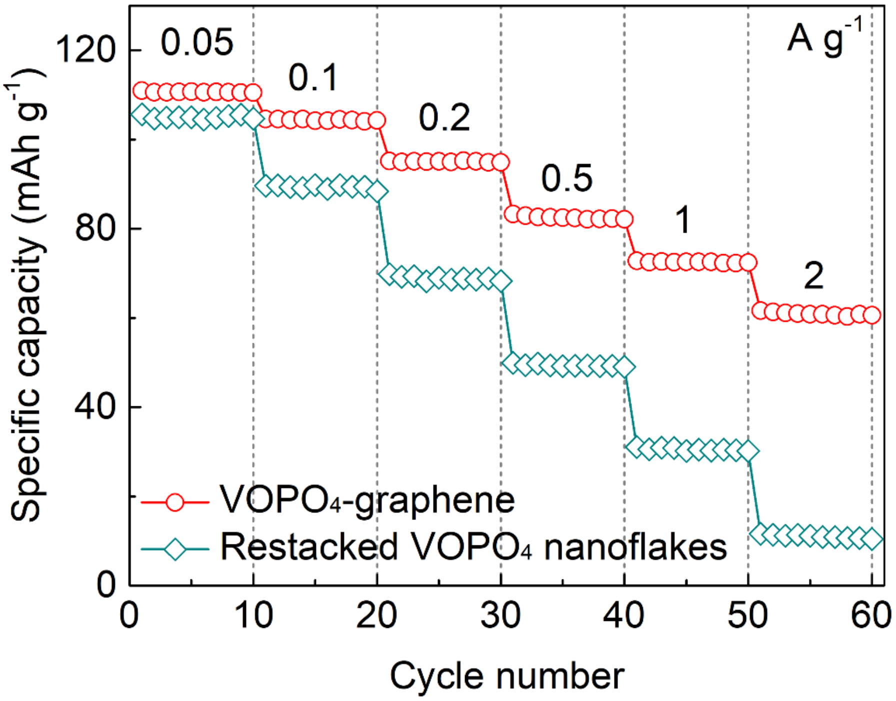

Supplementary Figure 31. Rate capability of restacked $VOPO_4$ nanoflakes and $VOPO_4$-graphene as cathodes for Zn-ion batteries at various current densities

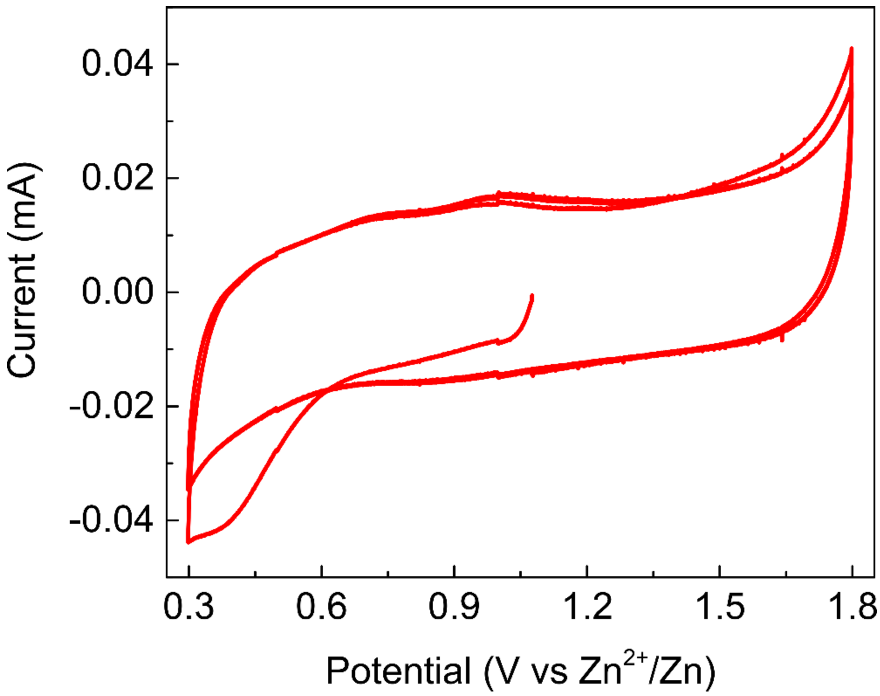

Supplementary Figure 32. CV curves of $VOPO_4$-graphene multilayered heterostructures as cathodes for Zn-ion batteries at 0.1 mV s^{-1}.

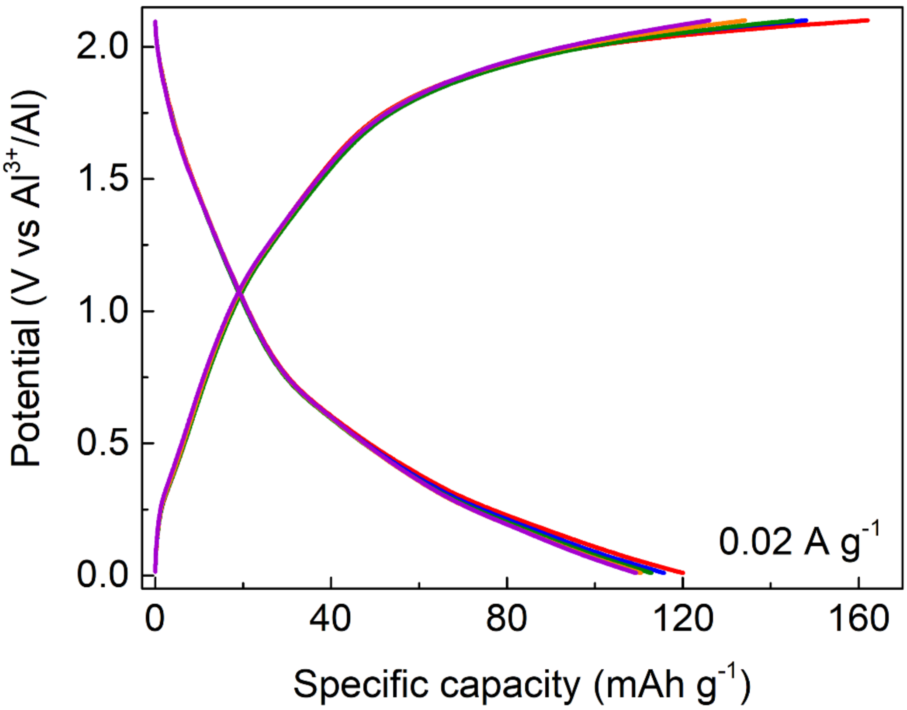

Supplementary Figure 33. Charge/discharge profiles of $VOPO_4$-graphene as cathodes for Al-ion batteries.

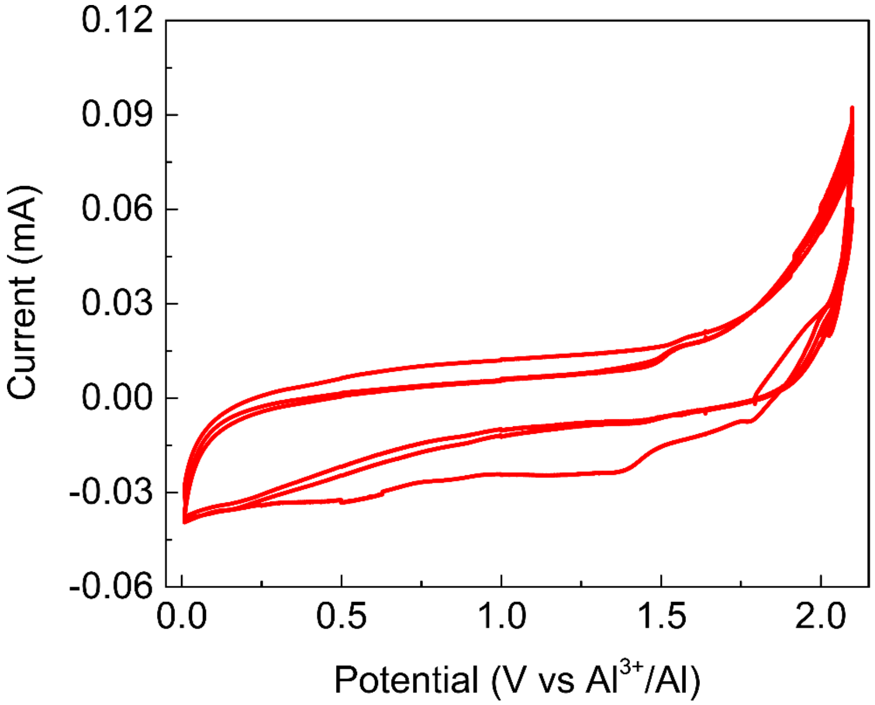

Supplementary Figure 34. CV curves of $VOPO_4$-graphene multilayered heterostructures as cathodes for Al-ion batteries at 0.1 mV s^{-1}.

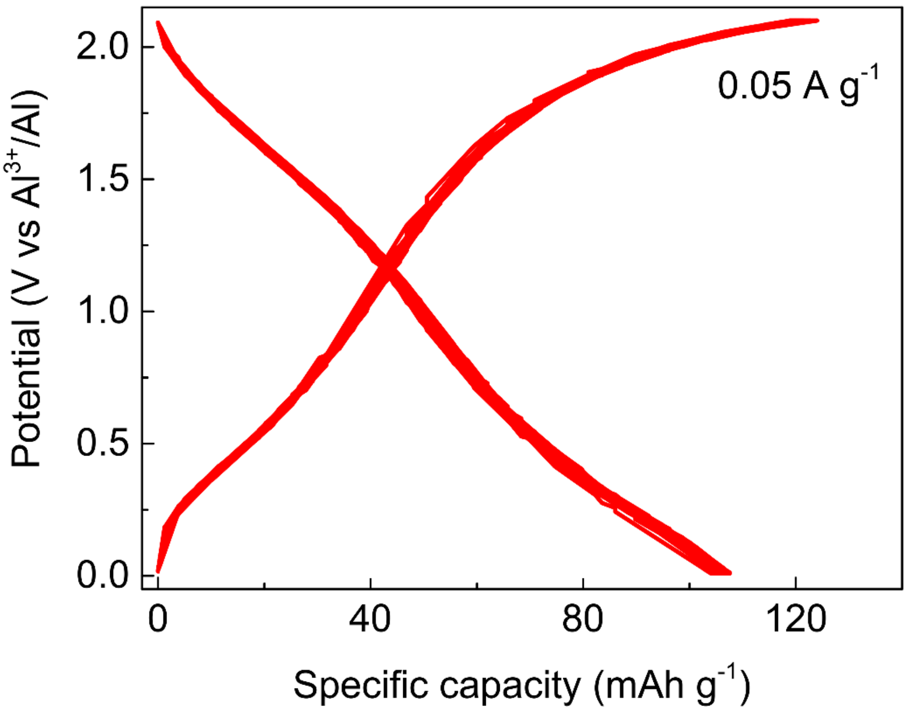

Supplementary Figure 35. Charge/discharge profiles of $VOPO_4$-graphene as cathodes for Al-ion batteries during the 100 cycles.

Supplementary Tables

Supplementary Table 1. Comparison of reported cathode materials for PIBs with the new 2D multilayered heterostructure cathodes in this work. The achieved energy densities were obtained based on the attained capacities and the average voltage of the respective cathode materials.

**Cathode materials**	**K-half cell electrolyte**	**Attained capacity mAh g^{-1}**	**Average voltage V vs K^+/K**	**Energy density Wh Kg^{-1}**	**Ref**
P3-$K_{0.5}MnO_2$	0.7 M KPF_6 in EC/DEC (1:1 vol%)	100	2.5 ~2.7 (2.6)	260	1
$K_{0.3}MnO_2$	1.5 M KFSI in EC/DMC	136	2.7	367.2	2
P2-$K_{0.6}CoO_2$	0.7 M KPF_6 in EC/DEC (1:1 vol%)	80	2.7	216	3
P2-$K_{0.65}Fe_{0.5}Mn_{0.5}O_2$	0.9 M KPF_6 in EC/DEC (1:1 vol%)	151	~2.5	377.5	4
$K_{2/3}Ni_{1/6}Co_{1/6}Mn_{2/3}O_2$	0.8 M KPF_6 in EC/DEC (1:1 vol%)	76.5	3.1	237.15	5
P2-$K_{0.41}CoO_2$	1 M KFSI in EC/DEC (1:1 vol%)	60	3	180	6
$K_2MnFe(CN)_6$	Saturated $KClO_4$ in PC	142	3.6	511.2	7
Prussian white (PW)	0.8 M KPF_6 in PC: 4wt % FEC	110	3.2	352	8
$KVPO_4F$	1 M KPF_6 in EC/PC	92	4.02	369.84	9

$K_3V_2(PO_4)_2F_3$	1 M KPF_6 in EC/PC	104	~3.7	385	10
$K_3V_2(PO_4)_3$	0.8 M KPF_6 in EC/DEC (1:1 vol%)	54	3.7	199.8	11
$KVOPO_4$	1 M KPF_6 in EC/PC	84	3.95	331.8	9
$KVOPO_4$	0.5 M KPF_6 in PC/FEC (1:1 vol%)	115	3.65	420	12
KVP_2O_7	0.5 M KPF_6 in EC/DEC	60	4.2	252	13
$K_2Ni_2TeO_6$	0.5 M KTFSI in Pyr_{13}TFSI	70	3.6	252	14
$VOPO_4$-graphene	1 M KPF_6 in EC/PC: 5wt % FEC	160	3.5	565	This work

Supplementary References

1. Kim, H. et al. Investigation of potassium storage in layered P3-type $K_{0.5}MnO_2$ cathode. *Adv. Mater.* **29**, 1702480 (2017).

2. Vaalma, C., Giffin, G. A., Buchholz, D. & Passerini, S. Non-aqueous K-ion battery based on layered $K_{0.3}MnO_2$ and hard carbon/carbon black. *J. Electrochem. Soc.* **163**, A1295−A1299 (2016).

3. Kim, H. et al. K-ion batteries based on a P2-type $K_{0.6}CoO_2$ cathode. *Adv. Energy Mater.* **7**, 1700098 (2017).

4. Deng, T. et al. Layered P2-type $K_{0.65}Fe_{0.5}Mn_{0.5}O_2$ microspheres as superior cathode for high-energy potassium-ion batteries. *Adv. Funct. Mater.* **28**, 1800219 (2018).

5. Liu, C. et al. $K_{0.67}Ni_{0.17}Co_{0.17}Mn_{0.66}O_2$: a cathode material for potassium-ion battery. *Electrochem. Commun.* **82**, 150−154 (2017).

6. Hironaka, Y., Kubota, K. & Komaba, S. P2- and P3-K_xCoO_2 as an electrochemical potassium intercalation host. *Chem. Commun.* **53**, 3693−3696 (2017).

7. Xue, L. et al. Low-cost high-energy potassium cathode. *J. Am. Chem. Soc.* **139**, 2164−2167 (2017).

8. He, G. & Nazar, L. F. Crystallite size control of prussian white analogues for nonaqueous potassium-ion batteries. *ACS Energy Lett.* **2**, 1122−1127 (2017).

9. Chihara, K., Katogi, A., Kubota, K. & Komaba, S. $KVPO_4F$ and $KVOPO_4$ toward 4 volt-class potassium-ion batteries. *Chem. Commun.* **53**, 5208−5211 (2017).

10. Lin, X., Huang, J., Tan, H., Huang, J. & Zhang, B. $K_3V_2(PO_4)_2F_3$ as a robust cathode for potassium-ion batteries. *Energy Storage Mater.* **16**, 97−101 (2019).

11. Han, J. et al. Investigation of $K_3V_2(PO_4)_3$/C nanocomposites as high-potential cathode materials for potassium-ion batteries. *Chem. Commun.* **53**, 1805−1808 (2017).

12. Liao, J. et al. Competing with other polyanionic cathode materials for potassium-ion batteries via fine structure design: new layered $KVOPO_4$ with a tailored particle morphology. *J. Mater. Chem. A* **7**, 15244−15251 (2019).

13. Park, W. B. et al. KVP_2O_7 as a robust high-energy cathode for potassium-ion batteries: pinpointed by a full screening of the inorganic registry under specific search conditions. *Adv. Energy Mater.* **8**, 1703099 (2018).

14. Masese, T. et al. Rechargeable potassium-ion batteries with honeycomb-layered tellurates as high voltage cathodes and fast potassium-ion conductors. *Nat. Commun.* **9**, 3823 (2018).
